# Hospital stay in patients admitted for acute bipolar manic episodes prescribed quetiapine immediate or extended release: a retrospective non-interventional cohort study (HOME)

**DOI:** 10.1186/s12888-014-0246-3

**Published:** 2014-08-31

**Authors:** Oğuz Karamustafalıoğlu, Andreas Reif, Murad Atmaca, Domingo Gonzalez, Miriam Moreno-Manzanaro, Miguel Angel Gonzalez, Esteban Medina, Antonello Bellomo

**Affiliations:** Department of Psychiatry, Şişli Etfal Teaching and Research Hospital, Istanbul, Turkey; Department of Psychiatry, Psychosomatics and Psychotherapy, Head Psychiatric Neurobiology and Bipolar Disorder Program, University of Würzburg, Würzburg, Germany; Department of Psychiatry, Firat University School of Medicine, Elazig, Turkey; Assertive Outreach, Birmingham & Solihull Mental Health Trust, Birmingham, UK; AstraZeneca Farmaceutica Spain, Madrid, Spain; Quintiles, Parque Empresarial Cristalia, Madrid, Spain; Department of Clinical and Experimental Sciences, Section of Psychiatry and Clinical Psychology, University of Foggia, Foggia, Italy

**Keywords:** Bipolar disorder, Acute mania, Quetiapine, Hospitalisation, Length of stay

## Abstract

**Background:**

Bipolar manic episodes often require hospital admission to ensure patient safety. The antipsychotic quetiapine is a common treatment for bipolar mania and is available in immediate release (IR) and extended release (XR) formulations; however, outcomes in patients receiving these different formulations have not been directly compared in an acute hospital setting.

**Methods:**

We conducted a multinational, observational, retrospective cohort study to describe and compare hospital stay in patients admitted for an acute bipolar manic episode treated with quetiapine IR or XR from 1 October 2009–1 October 2010. The primary outcome measure was comparison of length of stay (LOS) using zero-truncated negative binomial regression.

**Results:**

In total, 1230 patients were included (659 in the IR cohort; 571 in the XR cohort). The median LOS (interquartile range) was 18.0 days (12.0, 28.0) in the IR cohort and 20.0 days (12.0, 34.0) in the XR cohort, respectively. LOS was not significantly associated with quetiapine formulation irrespective of whether or not clinical characteristics were taken into account (p = 0.820 and p = 0.386, respectively). Overall, 84.2% and 84.4% of patients in the IR and XR cohorts, respectively, had not previously used quetiapine; of these patients, 78.7% and 68.9% received one total daily dose, and 14.4% and 23.9% received dose titration. Over half of patients received antipsychotic monotherapy (53.1% and 58.3% in the IR and XR cohorts, respectively) and most received a daily quetiapine dose ≥ 400 mg (64.9% and 71.8%, respectively, for quetiapine monotherapy and 59.9% and 80.3%, respectively, for combination treatment).

As a secondary outcome, multivariate analysis was used to identify other factors that affect LOS. Factors associated with a longer hospital stay included public funding versus private, maximum number of new medications administered, did not receive lithium and did not receive anxiolytics, sedatives/hypnotics (all p < 0.0001). Factors associated with a shorter hospital stay included presence of drug/alcohol abuse, living accompanied and having a psychiatric medical history (all p < 0.05).

**Conclusions:**

LOS was not found to be associated with quetiapine formulation. However, most patients received only one total daily dose of quetiapine without dose titration, which was unexpected and contrary to current recommendations.

**Trial registration:**

Trial registration: NCT01239589

**Electronic supplementary material:**

The online version of this article (doi:10.1186/s12888-014-0246-3) contains supplementary material, which is available to authorized users.

## Background

Bipolar disorder (BD) is a serious mood disorder characterised by the occurrence of one or more manic, hypomanic, depressive or mixed episodes [1]. The disorder has a significant negative impact on overall well-being and social, occupational and general functioning [2]. BD has a high global burden and is considered the fifth most disabling disease among adults aged 15–44 years in terms of years of life lived with a disability [3]. Patients who have experienced a mood episode are likely to suffer recurrences, with the probability of a recurrence during the year after recovery from an episode reported as more than 50%, increasing to more than 90% over 5 years [4]. The consequences of recurrent illness for patients and healthcare providers are substantial; BD is associated with an estimated annual direct healthcare cost of £487 per person in Europe, with direct non-medical costs of £468 and indirect costs of £6663 [5].

Manic episodes are specific to BD type I (BD-I), which has an estimated lifetime prevalence of 0.3–1.5% in Europe [6]. Acute bipolar manic episodes often constitute medical emergencies requiring admission to hospital to promote rapid recovery. Drug therapy is central to the management of acute mania, with mood stabilisers, typical antipsychotics and atypical antipsychotics comprising the most common treatment options [7,8].

Quetiapine is an atypical antipsychotic that has been shown to be effective and is licenced for the treatment of severe and acute bipolar mania [9,10]. Two formulations are available: quetiapine immediate release (IR) and quetiapine extended release (XR). Quetiapine IR is given twice daily and requires dose titration over 4 days until the target therapeutic dose is achieved, whereas quetiapine XR is given once daily and the dose titration is over 2 days. In the treatment of acute manic episodes, simplified dosing and a faster titration schedule may result in more rapid improvement, which might be expected to lead to a shorter duration of hospital stay.

The European study to describe *HO*spital stay in patients admitted for acute bipolar *M*anic *E*pisodes (HOME) was undertaken to compare the length of hospital stay (LOS) in patients treated with quetiapine IR or quetiapine XR. An observational study design was chosen to provide real-world data based on current clinical practice.

## Methods

### Study design and population

This was a multinational, multicentre, observational, cohort study (NCT01239589) of patients admitted to hospital for an acute bipolar manic episode treated with quetiapine IR or quetiapine XR during a fixed retrospective period (1 October 2009 to 1 October 2010). The patient cohorts were identified and data were collected between November 2010 and March 2011. The study was undertaken at 97 participating sites across 8 European countries (Belgium, Croatia, Denmark, Finland, Germany, Italy, Turkey and the UK).

Patients included in the analysis fulfilled the following criteria: diagnosed with BD (International Classification of Diseases – tenth revision); aged ≥ 18 years; admitted for an acute bipolar manic episode during the retrospective period; and treated with quetiapine XR or quetiapine IR at effective doses for manic episodes during the hospitalisation period (either during acute treatment or within 48 hours of continued hospitalisation). In patients with more than one acute manic episode during the retrospective period, the latest episode was considered as the study episode, which must have occurred 3 months after either response or remission of any previous manic episode.

Patients were excluded if: they had started hospitalisation before the study episode occurred for reasons other than a manic episode; the main reason for their hospitalisation was not BD; they were permanently hospitalised for BD; they were admitted for acute bipolar mania episodes but finally diagnosed with another type of episode; or they had received both quetiapine IR and XR during the same hospitalisation period. Patients who died during hospitalisation, those who were participating in a clinical trial during the hospitalisation period or who were pregnant were also excluded from analyses.

Investigators agreed a pre-set target of patient enrolment per study cohort (received quetiapine IR or XR). All identified patients who fulfilled the study inclusion criteria during the retrospective period were listed in an electronic web-based data capture system. If the number of eligible patients exceeded the target number, a computer program randomly selected a sample of patients to be enrolled in the study. Data for these randomly selected patients were collected from the hospital clinical records and recorded in the case report forms by the investigators during the period from 12 November 2010 to 1 March 2011.

### Ethics

This study was performed in accordance with ethical principles consistent with the Declaration of Helsinki, the International Conference on Harmonisation of Technical Requirements for Registration of Pharmaceuticals for Human Use and Good Clinical Practice. Approval was obtained from the relevant Ethics Committees according to local regulations, as detailed at the end of the manuscript. Due to the retrospective nature of the study, there was a likelihood of biasing the results due to a high non-response rate; therefore informed consent was obtained only in countries where this was a requirement. For countries where consent was required to use patient data (Denmark, Croatia, Italy, Germany), only data for patients with documented consent provided were included.

### Study objectives

The primary study objectives were to describe the hospital stay in patients admitted for an acute bipolar manic episode who were treated with either quetiapine IR or XR and to estimate and compare the LOS in these patients.

Secondary study objectives were: to describe demographic and other patient-related factors that could be linked to the use of quetiapine IR and XR; assessment of factors related to LOS in both cohorts; estimation of the difference in adjusted number of in-hospital days for specific subgroups of patients; and estimation of differences in use of hospital resources between the two cohorts during their hospital stay.

### Sample size

A sample size of 500 per group was calculated to provide an estimated 86.4% statistical power to show meaningful differences in LOS, provided that the mean and standard deviation (SD) LOS were similar to those found in a previous study with similar outcome measures (mean [SD] of 8.75 [12.18] days in the quetiapine IR group compared with 6.91 [5.70] days in the quetiapine XR group) [11].

### Statistical analysis

The analysis population comprised all eligible patients enrolled into the study for whom relevant data were available. All statistical analyses were performed using the statistical software system SAS Version 9.2 [12]. Results were considered significant at the 5% level.

Descriptive statistics (without statistical tests) were used to assess demographic and patient-related factors (e.g. medical history, disease characteristics, medications received prior to and during hospitalisation) that could be linked to quetiapine use.

The primary outcome of LOS was defined as the date of discharge minus the date of admission, presented as medians with interquartile ranges. A prespecified, two-step analysis was undertaken to determine any differences in LOS between the cohorts: 1) a generalised linear model was fitted to the data, using the number of days as the response, and treatment and country as explanatory variables; 2) zero-truncated negative binomial regression analysis was performed. This two-step analysis was also repeated with various demographic and clinical characteristics included as explanatory variables (prespecified). Sensitivity analyses were then used to assess the impact of the selected data transformation method (using prespecified log-normal regression analysis) and the impact of outliers (using prespecified zero-truncated negative binomial regression analysis) on the LOS.

Propensity score methodology (Additional file 1) was used as an additional exploratory analysis for the comparison of LOS between the two cohorts.

For secondary outcomes, factors associated with LOS were determined using a sequential approach. Firstly, univariate analyses of LOS with treatment, country and additional demographic and clinical characteristics were performed using zero-truncated negative binomial regression. Variables with a p-value < 0.2 were considered for inclusion in the multivariate analysis. Secondly, multivariate analysis was performed on the factors identified in the first step and other possible confounders and/or clinically significant factors, with the LOS as the response (Additional file 1).

## Results

### Baseline demographics and clinical characteristics

**Figure 1:**
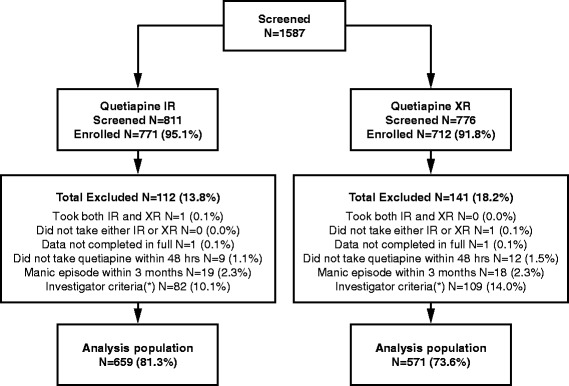
**Disposition of patients.** IR, immediate release; XR, extended release; (*) Due to patient not fulfilling all the inclusion/exclusion criteria.

A total of 1587 subjects were screened for study participation (Figure 1). Of these, 1483 were enrolled into the study (771 in the quetiapine IR cohort and 712 in the quetiapine XR cohort). A total of 1230 patients were eligible for inclusion in the analysis population (659 and 571 in the quetiapine IR and XR cohorts, respectively). The number of patients included in the analysis population by country was: 47 (Belgium); 75 (Croatia); 8 (Denmark); 65 (Finland); 152 (Germany); 219 (Italy); 499 (Turkey); and 165 (UK). The main reason for exclusion from the analysis population was failure to meet study inclusion/exclusion criteria in both cohorts.

Baseline demographics, medical history and disease characteristics were similar in the two cohorts (Table 1). The proportion of patients living accompanied was 78.8% and 73.7% in the quetiapine IR and XR cohorts, respectively, and a family history of mental illness was found in 28.1% and 33.1% of patients in the quetiapine IR and XR cohorts, respectively. The severity of the manic event leading to hospitalisation was similar in the two cohorts (severe in 64.6% of patients in the quetiapine IR cohort and 64.5% of those in the quetiapine XR cohort).

**Table 1:** **Baseline demographics and disease characteristics**

	**Quetiapine IR**	**Quetiapine XR**	**Total**
**Characteristic**	**(N = 659)**	**(N = 571)**	**(N = 1230)**
Gender (male), N (%)	337 (51.1)	279 (48.9)	616 (50.1)
Age (years), mean (SD)	42.8 (13.7)	42.4 (13.3)	42.7 (13.5)
Years in education, mean (SD)	9.9 (3.9)	10.3 (3.9)	10.1 (3.9)
Currently employed, N (%)	178 (27.0)	164 (28.7)	342 (27.8)
Cohabitation (lives accompanied), N (%)	519 (78.8)	421 (73.7)	940 (76.4)
Alcohol abuse/dependence,* N (%)	113 (17.1)	104 (18.2)	217 (17.6)
Drug abuse/dependence,* N (%)	46 (7.0)	44 (7.7)	90 (7.3)
Any relevant psychiatric medical history, N (%)	75 (11.4)	53 (9.3)	128 (10.4)
Any relevant non-psychiatric medical history, N (%)	158 (24.0)	134 (23.5)	292 (23.7)
Family history of mental illness, N (%)	190 (28.8)	189 (33.1)	379 (30.8)
Time since first diagnosis to hospital admission (years), median	5.7	6.3	5.9
Episode polarity diagnosis, N (%)			
Manic	453 (68.7)	352 (61.6)	805 (64.5)
Hypomanic	18 (2.7)	25 (4.4)	43 (3.5)
Depressive	86 (13.1)	63 (11.0)	149 (12.1)
Mixed	36 (5.5)	25 (4.4)	61 (5.0)
No other specified	5 (0.8)	5 (0.9)	10 (0.8)
Unknown	61 (9.3)	98 (17.2)	159 (12.9)
Previous hospital admission due to bipolar disorder, N (%)	183 (27.8)	160 (28.0)	343 (27.9)
Any suicide attempt since diagnosis, N (%)	85 (12.9)	74 (13.0)	159 (12.9)
Severity of index manic event, N (%)			
Mild	22 (3.3)	17 (3.0)	39 (3.2)
Moderate	185 (28.1)	155 (27.1)	340 (27.6)
Severe, without psychotic symptoms	178 (27.0)	166 (29.1)	344 (28.0)
Severe, with psychotic symptoms	248 (37.6)	202 (35.4)	450 (36.6)
Unavailable	26 (3.9)	31 (5.4)	57 (4.6)

IR, immediate release; XR, extended release; SD, standard deviation.

*At time of hospitalisation.

Prior to admission (i.e. from the onset of first symptoms of the study episode to hospitalisation), just over a quarter of patients received some form of medication (27.9% of the quetiapine IR cohort and 28.2% of the quetiapine XR cohort). The types of medications taken by patients in both cohorts during this period were similar. Prior to hospitalisation, 103 patients (15.6%) in the quetiapine IR cohort were taking quetiapine IR (1 patient [0.2%] in the quetiapine IR cohort was taking quetiapine XR) and 87 patients (15.2%) in the XR cohort were taking quetiapine XR (2 patients [0.4%] in the quetiapine XR cohort were taking quetiapine IR).

### Primary outcomes

#### LOS

**Figure 2:**
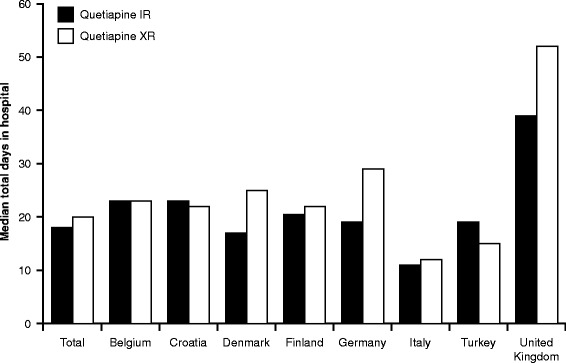
**Length of hospital stay (LOS).** IR, immediate release; XR, extended release.

The median (interquartile range) duration of hospitalisation was 18.0 (12.0, 28.0) days for the quetiapine IR cohort and 20.0 (12.0, 34.0) days for the quetiapine XR cohort. The analysis of LOS adjusted for cohort and country alone showed no statistically significant differences in LOS between the two cohorts (p = 0.820). This was substantiated by the sensitivity analyses of the impact of selected data transformation method (p = 0.125) and of the impact of outliers on the LOS (p = 0.213), which showed no evidence of a difference between the two cohorts. The analysis of LOS adjusted for cohort, country and clinical characteristics also showed no evidence of a difference between cohorts (p = 0.386). Patients in the UK had the longest median LOS compared with any other country (48.0 days compared with 26.0 for Germany, 23.0 for Belgium, 17.0 for Denmark, 22.0 for Croatia, 21.0 for Finland, 18.0 for Turkey and 11.0 days for Italy) (Figure 2).

#### Estimated LOS analysed by propensity score

Using logistic regression, propensity groups were derived based on the predicted probability of cohort membership. Factors related to prescription of quetiapine XR were: type of hospital, size of hospital, prescription of quetiapine from first symptoms of the study episode, and the time since diagnosis. There was no difference in LOS adjusted for the propensity score either as a continuous covariate (p = 0.439) or as a categorical fixed effect (p = 0.115). Furthermore, there was no difference in LOS between cohorts in matching analysis (254 matched pairs of patients) (p = 0.752).

#### Description of hospital stay

The majority of patients were enrolled in regional/national hospitals (61.2% compared with 22.7% in general/district hospitals, 12.0% in rural/local/community hospitals and 4.1% from other types of hospital). More patients received quetiapine XR compared with quetiapine IR at rural/local/community hospitals (17.2% vs 7.4%), while the percentage of patients who received quetiapine IR and quetiapine XR at general/district (24.1% and 21.0%, respectively) and regional/national hospitals (65.1% and 56.7%, respectively) was similar. The majority of patients were enrolled from publically funded hospitals (92.1%), which corresponded to 91.5% of patients in the quetiapine IR cohort and 92.8% of patients in the quetiapine XR cohort.

As shown in Table 2, the majority of patients were admitted to psychiatric wards (82.4%). The percentage of patients admitted to psychiatric wards was similar in the two cohorts (82.5% in the quetiapine IR cohort and 82.1% in the quetiapine XR cohort). The mean ± SD number of different ward types in which patients stayed while hospitalised was similar between the two cohorts (1.2 ± 0.56 in the quetiapine IR cohort and 1.3 ± 0.59 in the quetiapine XR cohort).

**Table 2:** **Description of hospital stay**

	**Quetiapine IR**	**Quetiapine XR**	**Total**
**Characteristic**	**(N = 659)**	**(N = 571)**	**(N = 1230)**
Department of admission, N (%)			
Emergency room	84 (12.7)	52 ((9.1)	136 (11.1)
Intensive care unit	11 (1.7)	27 (4.7)	38 (3.1)
General ward	19 (2.9)	21 (3.7)	40 (3.3)
Psychiatric ward	544 (82.5)	469 (82.1)	1013 (82.4)
Other	1 (0.2)	2 (0.4)	3 (0.2)
Time from symptom onset to admission (days), median	8.0	8.0	8.0
No. of nights in ward, median			
Emergency room	1.0	1.0	1.0
Intensive care unit	5.5	18.0	14.0
General ward	13.0	20.5	15.0
Psychiatric ward	18.0	19.0	18.0
Other	5.0	10.5	9.0
Discharge status, N (%)			
Discharged to home	629 (95.4)	531 (93.2)	1160 (94.4)
Transferred to another facility	19 (2.9)	27 (4.7)	46 (3.7)
Other	9 (1.4)	10 (1.8)	19 (1.5)
Unknown	2 (0.3)	2 (0.4)	4 (0.3)

IR, immediate release; XR, extended release.

Data on new medication prescriptions during hospitalisation were collected to assess any treatment differences between the cohorts (summaries of new medications did not include those prescribed before hospital admission). In total, 97.0% of patients received medication during hospitalisation. Antipsychotics (including quetiapine) were the most commonly newly prescribed medication (94.9% of patients, [quetiapine IR cohort, 95.0%; quetiapine XR cohort, 94.7%]; some patients were already receiving quetiapine at the time of admission), followed by anticonvulsants (50.0% of total [quetiapine IR cohort, 45.8%; quetiapine XR cohort, 54.8%]), anxiolytics, sedatives and hypnotics (35.7% of total [quetiapine IR cohort, 32.5%; quetiapine XR cohort, 39.4%]) and lithium (25.3% of total [quetiapine IR cohort, 27.2%; quetiapine XR cohort, 23.1%]). A minority of patients were prescribed antidepressants (5.0% of total [quetiapine IR cohort, 4.7%; quetiapine XR cohort, 5.4%]), antiparkinsonians (4.5% of total [quetiapine IR cohort, 3.9%; quetiapine XR cohort, 5.1%]) and other medications (4.1% of total [quetiapine IR cohort, 3.9%; quetiapine XR cohort, 4.4%]).

Overall, 84.2% and 84.4% of patients in the IR and XR cohorts, respectively, had not previously used quetiapine. Of these patients that were receiving quetiapine for the first time, most received only one total daily dose of quetiapine (quetiapine IR cohort: 78.7%; quetiapine XR cohort: 68.9%), and only 14.4% and 23.9% of patients, respectively, received the recommended dose titration during the first 7 days of hospitalisation. The first in-hospital quetiapine mean dosage was 447.7 mg for the quetiapine IR cohort and 482.4 mg for the quetiapine XR cohort. The last in-hospital quetiapine mean dosage was 504.7 mg for the quetiapine IR cohort and 561.4 mg for the quetiapine XR cohort. The mean change in quetiapine dosage (first to last) was 57.0 mg for the quetiapine IR cohort and 78.9 mg for the quetiapine XR cohort. Quetiapine was given as antipsychotic monotherapy in 53.1% of patients in IR cohort and 58.3% of patients in the XR cohort. The majority of patients received a daily dose of quetiapine ≥400 mg (patients receiving quetiapine alone: 64.9% in the quetiapine IR cohort and 71.8% in the quetiapine XR cohort, respectively; patients receiving quetiapine in combination with other antipsychotics: 59.9% in the quetiapine IR cohort and 80.3% in the quetiapine XR cohort, respectively).

### Secondary outcomes

#### Factors related to LOS

Multivariate analysis identified several clinical factors related to the total LOS (Table 3). Variables associated with a longer hospital stay were: public hospital funding, hospital admission related to BD in the previous 12 months, not experiencing a manic episode in the previous 12 months, a severe versus a mild manic episode, admission from a psychiatric ward versus emergency room, maximum number of new medications administered, not being treated with lithium during the hospital stay, and not being treated with anxiolytics/sedatives/hypnotics during the hospital stay. The variables associated with a shorter LOS were: living accompanied, presence of alcohol/drug abuse/dependence and having a psychiatric medical history.

**Table 3:** **Factors associated with the total length of hospital stay (LOS)**

	**Estimate (95% CI)**	**t-statistic**	**p-value**
**Site characteristics**			
**Public funding vs private**	**1.442 (1.259, 1.651)**	**5.28**	**< 0.0001**
Less than 50 beds vs more than 600 beds	1.015 (0.851, 1.212)	0.17	0.8651
50 to 200 beds vs more than 600 beds	0.936 (0.846, 1.035)	−1.29	0.1966
201 to 600 beds vs more than 600 beds	1.011 (0.923, 1.107)	0.24	0.8101
Patient demographics			
**Lives accompanied vs alone**	**0.889 (0.812, 0.974)**	**−2.54**	**0.0112**
**Any present alcohol/drug abuse or dependence**	**0.883 (0.805, 0.968)**	**−2.66**	**0.0079**
Medical history			
**Any psychiatric medical history**	**0.868 (0.770, 0.978)**	**−2.33**	**0.0202**
Any non-psychiatric medical history	1.064 (0.980, 1.156)	1.48	0.1392
**Any BD hospital admission in prior 12 months**	**1.179 (1.071, 1.297)**	**3.37**	**0.0008**
**No manic event in prior 12 months**	**1.188 (1.076, 1.312)**	**3.41**	**0.0007**
Severity of manic event			
Moderate vs mild	0.931 (0.763, 1.136)	−0.71	0.4799
**Severe vs mild**	**1.250 (1.031, 1.514)**	**2.27**	**0.0231**
Department of admission			
ICU vs ER	0.991(0.782, 1.255)	−0.08	0.9397
General ward vs ER	1.236 (0.985, 1.551)	1.84	0.0667
**Psychiatric ward vs ER**	**1.180 (1.035, 1.345)**	**2.47**	**0.0137**
Treatment			
**Maximum no. of new medications administered**	**1.154 (1.122, 1.187)**	**10.00**	**< 0.0001**
	0.969 (0.903, 1.040)	−0.87	0.3861
**Did not receive lithium**	**1.211(1.107, 1.325)**	**4.18**	**< 0.0001**
**Did not receive anxiolytics, sedatives/hypnotics**	**1.222 (1.119, 1.334)**	**4.47**	**< 0.0001**
Received antidepressants	1.144 (0.971, 1.348)	1.61	0.1069
% quetiapine use	0.999 (0.996, 1.001)	−1.29	0.1970

BD, bipolar disorder; CI, confidence interval; ER, emergency room; ICU, intensive care unit.

Factors associated with a LOS are shown in bold. Estimates with 95% CI were calculated by zero-truncated negative binomial regression multivariable analysis. Only clinical factors included in the final model are shown. Countries were included in the model but are not shown. Clinical factors with a p-value of < 0.05 were considered significant.

#### Comparison of LOS in patient subgroups

Analysis of several pre-specified subgroups, adjusted for cohort and country, was performed to assess any association with the type of quetiapine received. The analyses suggested that none of the variables assessed were associated with the type of quetiapine received during hospitalisation (as determined by zero-truncated negative binomial regression).

#### Use of resources during hospitalisation

Service visits during the hospital stay included psychologists, group therapy, substance abuse counselling and social worker visits. Overall, there were no differences (as determined by logistic regression) in the incidence of resource use between the quetiapine IR and XR cohorts when all service visits were taken into account (odds ratio [OR] = 1.30, 95% confidence interval [CI]: 0.87, 1.94; p = 0.201). Similarly, there were no differences (as determined by logistic regression) between the cohorts in the incidence of laboratory tests (OR = 0.93, 95% CI: 0.34, 2.57; p = 0.890). Laboratory tests received during hospitalisation included blood/biochemistry tests, and echocardiograms.

## Discussion

The length of hospital admission is often used as a direct indicator of effectiveness of treatments for psychiatric disorders including BD. Over the past several decades, the LOS for patients requiring psychiatric hospitalisation has decreased from months to weeks, driven not only by economic pressures but by advances in clinical practice with a focus on treatment in outpatient settings [13–15]. There is some debate as to the optimum LOS in psychiatric patients; some studies argue that patients with depression who are discharged after a brief inpatient treatment are more depressed and more globally impaired on discharge [13], while other studies suggest that shorter stay is as effective or more effective than long-term inpatient programmes among depressed patients [16,17], or patients with severe mental illness [18].

This retrospective study was undertaken to describe and compare hospital stay in patients admitted for an acute bipolar manic episode treated with either quetiapine IR or quetiapine XR. The study was designed to obtain information on actual clinical practice and to better understand the unmet medical and health care needs in patients admitted to hospital with bipolar mania. We aimed to minimise selection bias through use of a retrospective design to avoid prescription induction and additional procedures, a random sampling process for study patient selection, and enrolment of a similar number of patients in each cohort.

In our study, the LOS was not found to be significantly associated with the quetiapine formulation received, irrespective of whether or not intervening factors were taken into account. There were also no differences in LOS when propensity score methodology was applied. It had been expected that the faster titration to therapeutic dosages possible with quetiapine XR might lead to faster resolution of symptoms and a shorter hospital stay in patients treated with this formulation compared with those who received quetiapine IR. However, the data collected revealed that in this population of acutely manic hospitalised patients, most were treated with only one total daily dose of quetiapine. Furthermore, only a minority of patients in both cohorts actually had a dose titration during the first 7 days of hospitalisation, with very little change from the first to the last in-hospital quetiapine dosage seen in either cohort, thus were not following the recommendation that quetiapine IR should be given twice daily with a 4-day dose titration. As a result, the presumed advantage of quetiapine XR is difficult to assess; this off-label use of IR may lead to different outcomes that are not captured by LOS. A numerically higher proportion of patients in the quetiapine XR cohort than in the quetiapine IR cohort were receiving antipsychotic monotherapy, which may warrant further research to explore whether quetiapine XR could be a more cost-effective treatment. The proportion of patients on quetiapine XR that reached a daily dose of ≥ 400 mg was numerically slightly higher than for patients on quetiapine IR, both as monotherapy and in combination with other antipsychotics. Factors found to be related to prescription of quetiapine XR were type of hospital, hospital size, XR prescription from first symptoms and time since diagnosis.

As expected, the majority of patients admitted to hospital for a manic bipolar episode received new prescriptions; antipsychotic prescriptions alone were received by 95% of patients. Somewhat surprisingly, 5% of patients received new prescriptions for antidepressants during hospitalisation. This observation is not only counter-intuitive, but also contradicts treatment guidelines which recommend ceasing antidepressant treatment at the onset of manic episodes [19]. Although several studies have investigated the role of antidepressants in the switch from depression to mania [20,21], and antidepressant use in bipolar mania has been recorded in other observational studies such as EMBLEM and WAVE-bd [8,22], we know of no controlled, randomised studies investigating the use of antidepressants during manic episodes. Based on our findings, we believe more research is needed to explore how widespread this clinical practice is and whether it confers any benefits to patients.

In our study, the median LOS was found to be 18.0 days for the quetiapine IR cohort and 20.0 days for the quetiapine XR cohort. This was higher than the LOS observed in a US study [11], which collected national data from an administrative database and was used to calculate the study sample size (average 8.75 days in the IR cohort and 6.91 days in the XR cohort). Similar LOS in bipolar manic patients prescribed quetiapine IR or XR were subsequently reported by a US study (least squares mean 9.6 days in the IR cohort vs 9.0 days in XR cohort) [23]. Our findings are more consistent with a European study, which found that patients with BD (all polarities) were hospitalised for a mean length of 18.1 days in Spain and 20.4 days in France [24]. The authors of this study noted that hospitalisations for patients with BD appeared to be shorter in the US compared with Europe, although the LOS in our study was found to differ among the participating countries, with patients in the UK having the longest median LOS compared with any other country. These differences among countries may reflect variation in clinical practice for the management of acute bipolar mania, differences in access to participating hospitals, and cultural factors influencing health-seeking behaviours.

LOS has been shown to be influenced by many factors including living conditions, access to community services and comorbidities [18,25,26]. Our study identified several factors related to LOS, including recent disease history (hospitalisation and experiencing a manic episode in the previous 12 months); degree of cohabitation; drug and alcohol abuse; severity of the episode; and several factors that may be influenced by the severity of the episode (number and type of new medications received, and department of admission). A similar study found that patients with a higher number of previous episodes were more likely to stay longer in hospital, and suggested that this is indicative of more severe illness [14].

Notably, not being treated with lithium or anxiolytics, sedatives and hypnotics during hospitalisation was associated with a longer hospital stay, which may suggest that these treatments could improve outcomes related to LOS in hospitalised manic patients. Inverse associations (lithium treatment related to a shorter LOS, and anxiolytic/sedative/hypnotic treatment related to shorter LOS) were not found. However, it should be noted that the duration of lithium use could be an influencing factor on LOS as, if treatment is started prior to (rather than during) hospitalisation, a more pronounced effect on the duration of hospitalisation may be seen. Another surprising finding was that alcohol/drug abuse at the time of hospitalisation was related to a shorter LOS. Alcohol abuse during hospitalisation may be due to self-medication as many patients discover the central nervous system depressant effects of alcohol and thus use it to decrease their anxiety and as an aid to induce sleep. Other studies assessing the effect of this factor on hospital stay are scarce and provide conflicting results; a retrospective study by Fan *et al.* found no significant difference in LOS between alcoholic and non-alcohol manic patients [27]. Alcohol withdrawal in hospital is a common cause of relapse, but also a cause of additional symptoms difficult to distinguish from mania (agitation, euphoria) that may lead to decreased LOS compared with a more pure acute manic episode. Further investigation is needed to establish the reason behind this result.

### Limitations

Data dispersion in LOS observed in this study was higher than expected according to the sample size estimation based on the Järbrink study [11], which may at least in part explain why statistically significant differences were not achieved. Limitations of the retrospective study design include the fact that the data were not originally collected according to a defined protocol, the potential for missing data, lack of documentation of disease severity and clinical outcomes using recognised clinical scales, and variability between different study sites. As this study utilised an observational design rather than a prospective randomised design, any probability values should be viewed with caution.

From a broader perspective of BD, it is important to note that this study only considered the treatment of acute manic episodes. However, morbidity from mania is not limited to acute episodes as full recovery of functioning often lags months behind remission of symptoms [28]. Maintenance of euthymia is an important goal in the clinical management of patients with BD and is best achieved through long-term drug therapy [29].

## Conclusions

In summary, this study was successful in describing the hospital stay and allowing comparisons in the management of patients admitted for an acute bipolar manic episode treated with either quetiapine IR or quetiapine XR. LOS was not found to be associated with the formulation of quetiapine received. However, most patients received only one total daily dose of quetiapine, without dose titration at initiation of treatment, which was unexpected and contrary to current recommendations.

## Additional file

Additional file 1:
**Statistical analysis.**

## Abbreviations

BD: Bipolar disorder
BD-I: BD type I
CI: Confidence interval
HOME: European study to describe hospital stay in patients admitted for acute bipolar manic episodes
IR: Immediate release
LOS: Length of stay
OR: odds ratio
SD: Standard deviation
XR: Extended release
