# Supplementary material for: Hospital stay in patients admitted for acute bipolar manic episodes prescribed quetiapine immediate or extended release: a retrospective non-interventional cohort study (HOME)
Source: BMC Psychiatry. 2014 Aug 31;14:246. doi: 10.1186/s12888-014-0246-3 (PMC4159508; doi:10.1186/s12888-014-0246-3)
Supplement: Additional file 1: — Statistical analysis. [file 12888_2014_246_MOESM1_ESM.docx]

# Additional file 1

## Statistical analysis

**Propensity score methodology**

The propensity score is the conditional probability of receiving either quetiapine IR or quetiapine XR during hospitalisation, based on a set of known covariates (patient characteristics). Propensity scores were derived with logistic regression. The propensity score was either included as a continuous covariate or as a categorical (quintiles) covariate for the analysis of LOS. For identification of factors related to length of stay (LOS), a similar sequential approach to that used for the primary endpoint was followed.

**Multivariate analysis of secondary outcomes**

The type of model used was prespecified, with characteristics included in the multivariate analysis based on the data. The characteristics considered in the model-fitting process included: site characteristics (type of hospital, type of funding, hospital size); patient demographics (sex, age, years of education, professional status, degree of cohabitation, current alcohol/drug dependence); medical history; disease characteristics (time since first diagnosis, polarity of the episode at diagnosis, number of hospital admissions, number of suicide attempts, number of disease events, time since previous event date); event information (severity of manic event, time from onset to admission, department of admission); and medication use at admission or during hospitalisation.

**Comparison of LOS in patient subgroups**

Estimated differences in the LOS in specific patient subgroups were analysed using zero-truncated negative binomial regression. LOS was compared between cohorts in the following subgroups- *prespecified*: patients receiving monotherapy; patients receiving polytherapy; type of hospital (rural/community hospitals, general/district hospitals, regional/national hospitals); patients admitted for over 60 days; *non-prespecified*: quetiapine dose of 400–800 mg; quetiapine dose
>400 mg; patients first admitted to a psychiatric ward. Estimated differences in the incidence of resource use between cohorts were assessed by logistic regression containing factors for cohort and country.
